# Triglycerides, Cholesterol, and Depressive Symptoms Among Undergraduate Medical Students: A Cross-Sectional Study

**DOI:** 10.3390/diseases13100326

**Published:** 2025-10-02

**Authors:** Maximiliano Olguín-Montiel, Alejandro Álvarez-Flores, Dulce Milagros Razo-Blanco-Hernández, María Alicia Mejía-Blanquel, Verónica Fernández-Sánchez, Gledy Manuela Olmos-Rivera, Ana Cristina Castañeda-Márquez, Edith Araceli Cano-Estrada, Mónica Alethia Cureño-Díaz, José Ángel Hernández-Mariano

**Affiliations:** 1 School of Medicine, National Autonomous University of Mexico, Mexico City 04510, Mexico; 2 Department of Research, Hospital Juarez of Mexico, Mexico City 07760, Mexico; dulce.razo@salud.gob.mx (D.M.R.-B.-H.);; 3 Faculty of Higher Studies Iztacala, National Autonomous University of Mexico, Tlalnepantla 54090, Mexico; 4 Nursing School, Hospital Juárez de México, Mexico City 04510, Mexico; 5 Scientific Research Institute, Juarez University of the State of Durango, Durango 34000, Mexico; anacristina.castaneda@ujed.mx; 6 Superior School of Tlahuelilpan, Autonomous University of the State of Hidalgo, Tlahuelilpan 42780, Mexico; 7 National Institute of Cancerology, Mexico City 14080, Mexico

**Keywords:** dyslipidemias, hypertriglyceridemia, hypercholesterolemia, depression, medical students

## Abstract

Background: Depression is one of the most common mental disorders among undergraduate students, particularly those in medical training, who face high academic demands and emotional burdens. Biological factors such as lipid abnormalities have been proposed as contributors to depressive symptoms, although evidence in this group is scarce. Therefore, we aimed to evaluate the association between triglyceride and total cholesterol levels and depressive symptoms in medical students. Methods: We conducted a cross-sectional study including 219 medical students from a public university in Mexico. Depressive symptoms were assessed using the CESD-7 scale, validated in the Mexican population. Fasting triglyceride and total cholesterol concentrations were measured with the Accutrend Plus analyzer. Prevalence ratios (PRs) and 95% confidence intervals (CIs) were estimated using robust Poisson regression, adjusting for potential confounders. Results: Overall, 38.8% of students presented depressive symptoms. In adjusted continuous models, each 10 mg/dL increase in triglycerides was associated with a 4% higher prevalence of depression (PR = 1.04, 95% CI 1.03–1.06), while each 10 mg/dL increase in total cholesterol was associated with a 13% higher prevalence (PR = 1.13, 95% CI 1.05–1.21). Analyses using clinically relevant cutoffs confirmed these associations: triglycerides ≥ 150 mg/dL (PR = 1.76, 95% CI 1.24–2.48) and cholesterol ≥ 200 mg/dL (PR = 1.66, 95% CI 1.19–2.31). Conclusions: Dyslipidemias may play a relevant role in the mental health of young adults and highlight the importance of incorporating metabolic risk assessment into strategies to prevent and address depression in medical students.

## 1. Introduction

Depression is one of the most common mental disorders worldwide. The World Health Organization (WHO) estimates that it affects approximately 332 million people globally and is one of the leading causes of disability-adjusted life years lost [1].

Undergraduate students are a particularly vulnerable population to mental health problems, as they operate in highly demanding academic environments. The combination of full-time courses, excessive workloads, and pressure to perform satisfactorily results in high levels of stress. Added to this are peer competition, high faculty expectations, lack of sleep, and anxiety about their professional future. These stresses, along with changes in social and family networks associated with the transition to adulthood, increase psychological susceptibility and promote the development of depressive symptoms in the university population [2,3].

Globally, the prevalence of depressive symptoms among undergraduate students has been estimated at approximately 33.6% [4]. Within this population, health science students appear particularly vulnerable, given the high academic and clinical demands, the early exposure to human suffering, and the pressure to develop professional competencies within a limited timeframe [5,6]. The most robust evidence comes from medical students, where reported prevalence rates of depression range between 28% and 48% [7,8] markedly higher than the estimated prevalence in the general population (5% according to WHO) [1].

Although depression has traditionally been examined primarily from a psychosocial perspective, growing attention has recently focused on the biological and metabolic determinants that may contribute to its onset. Among these, disturbances in lipid metabolism have emerged as potential risk factors for cardiovascular disease. Dyslipidemias, particularly hypercholesterolemia and hypertriglyceridemia, are relevant not only for their well-established role in cardiovascular disease but also for their possible implications in mental health [9].

Previous studies have documented a high prevalence of dyslipidemias among undergraduate students, with estimates ranging from 33.8% to 86.7% [10,11]. In medical students specifically, up to 41.8% have been reported to present low levels of high-density lipoprotein cholesterol (HDL-C), a key component of metabolic syndrome [12]. These findings suggest that this population is exposed to a dual burden combining the academic and emotional strain inherent to their training and the metabolic risks associated with cardiovascular disease.

Several biological mechanisms may explain the association between serum lipids and depression. One proposed pathway involves low-grade systemic inflammation, a hallmark of dyslipidemia, which has been linked to alterations in neurotransmitter systems such as serotonin and dopamine [13]. Furthermore, elevated triglyceride and cholesterol levels may modify neuronal membrane composition, reducing receptor fluidity and altering synaptic transmission [14].

In Mexico, depression is a significant public health concern among young people [15], while the prevalence of dyslipidemias has increased steadily in recent decades, including among young adults [16,17]. Although several studies have investigated the prevalence of depression in medical students [6,18,19,20], research on lipid alterations in this group remains scarce and is generally restricted to reports from broader university populations [21,22,23]. To the best of our knowledge, no study has been published either in Mexico or internationally that specifically examines the association between lipid profiles and depressive symptoms in medical students, underscoring a significant gap in the literature.

Generating evidence in this field is particularly relevant, as it would allow for a more comprehensive understanding of the risk factors faced by this highly vulnerable population. Furthermore, it would contribute to the design of preventive and health promotion interventions that integrate both academic–emotional and metabolic components, with the potential to reduce the long-term impact of depression and cardiovascular disease, and to strengthen institutional programs that promote both mental and metabolic health among medical students. Given the above, we aimed to evaluate the association between triglyceride and total cholesterol levels and the presence of depressive symptoms in medical students.

## 2. Materials and Methods

### 2.1. Design and Study Population

We conducted a cross-sectional analytical study between March to July 2025 among medical students at a public university in the State of Mexico. The sample size was determined using the finite population formula, considering that at the time of the study, 1223 students were enrolled in the program. Assuming an expected prevalence of depressive symptoms of 48% among medical students [8], a minimum sample size of 219 participants was calculated, with a 95% confidence level and a 6% margin of error. A simple random sampling technique was employed to select participants. Eligible students were men and non-pregnant women aged 18 years or older. Exclusion criteria included a previous diagnosis of thyroid disease, polycystic ovary syndrome, or current treatment with psychotropic medications or steroids. Of the 219 randomly selected potential participants, four were ineligible during pre-screening and were replaced by the following candidates from the pre-randomized list within the same sampling frame. Participation among eligible participants was 100%, yielding a final recruited and analyzed sample of *n* = 219. The eligibility rate among the initially invited candidates was 215/219 (98.2%). No missing data was observed in the questionnaires. The selection process is depicted in Supplementary Figure S1 (flowchart).

### 2.2. Data Collection

#### 2.2.1. Sociodemographic and Academic Variables

A general data questionnaire was applied to collect sociodemographic information, including age, sex, marital status, monthly family income, and living arrangements. Additionally, academic variables were collected, including the year of study and whether the student received a scholarship.

#### 2.2.2. Substance Use, Comorbidities, and Family History

Questions were included regarding current tobacco and alcohol use, defined as any consumption within the past 30 days. Students were also asked about their medical diagnoses of chronic diseases that require routine pharmacological treatment. In addition, parental history of depression and overweight/obesity was recorded and coded as dichotomous variables (yes/no).

#### 2.2.3. Reproductive Health Variables

All students were asked about contraceptive use. Among women, additional questions inquired about the use of hormonal contraceptive methods and the presence of menstrual cycle irregularities.

#### 2.2.4. Anthropometric Measurements

Body weight was measured using a portable Tanita© scale (Tanita Corp, Tokyo, Japan) with a reading precision of 0.2 kg for individuals weighing ≤ 100 kg and 0.4 kg for those between 100 and 200 kg. The scales were calibrated daily before use. Height was measured in centimeters (cm) with a portable SECA© stadiometer (SECA, Hamburg, Germany) with a reading precision of 1 mm. Body mass index (BMI) was calculated as weight in kilograms divided by height in meters squared (kg/m^2^). For analytical purposes, BMI was classified as normal (<24.9 kg/m^2^) and overweight/obesity (≥25.0 kg/m^2^) [24].

#### 2.2.5. Depressive Symptoms

Depressive symptoms were assessed using the seven-item shortened version of the Center for Epidemiologic Studies Depression Scale (CESD-7). This instrument, validated in the Mexican population, has demonstrated good internal consistency (Cronbach’s alpha > 0.83). It consists of seven items rated on a four-point Likert scale, with total scores ranging from 0 to 21. A cut-off score of ≥5 was used to indicate the presence of depressive symptoms [25].

#### 2.2.6. Triglycerides and Total Cholesterol

Capillary blood samples were collected after a minimum of 8 h of fasting. Triglyceride and total cholesterol levels were measured using the portable Accutrend^®^ Plus analyzer (Roche Diagnostics, Mannheim, Germany), following the manufacturer’s recommendations. Previous studies in adult populations have demonstrated good reproducibility and high concordance of this device with standard laboratory methods for triglycerides and total cholesterol, although with a slight tendency to overestimate values [26]. Furthermore, comparative evaluations of total cholesterol self-tests have shown that the Accutrend^®^ Plus provides the best diagnostic performance, with 92% sensitivity and 89% specificity [27]. Given its portability, lower cost compared with laboratory assays, and acceptable diagnostic accuracy, this system represents a valid and feasible alternative for field-based monitoring of metabolic and cardiovascular risk factors. In this study, lipid measurements were limited to triglycerides and total cholesterol due to feasibility and resource constraints; measurements of HDL and LDL cholesterol were not conducted. For our primary analyses, we modeled triglycerides and cholesterol as continuous variables. In secondary analyses, and based on international clinical guidelines, triglyceride levels were categorized as normal (<150 mg/dL) or high (≥150 mg/dL), and total cholesterol as normal (<200 mg/dL) or high (≥200 mg/dL) [28].

#### 2.2.7. Data Collection Procedure

All data collection procedures were conducted in a specially designated classroom within the university, between 8:00 and 10:00 a.m. The process was carried out in two phases. In the first phase, participants were selected, informed consent forms were obtained, and questionnaires were administered to them. The following day, students were asked to attend after an overnight fast of at least 8 h for capillary blood sample collection. All measurements were performed by trained personnel, following standardized protocols to ensure the quality, accuracy, and reliability of the data collected. Capillary blood samples were collected under biosafety conditions, and all biological waste was managed and disposed of in accordance with national regulations.

### 2.3. Statistical Analysis

The characteristics of the study sample were summarized using frequencies and percentages for categorical variables, and medians with interquartile ranges for continuous variables due to their non-normal distribution as indicated by the Shapiro–Wilk test. Comparisons of lipid concentrations and categories according to depressive symptom status were performed using the Mann–Whitney U test and Pearson’s chi-square test, respectively.

To examine the association between triglyceride and total cholesterol concentrations and the presence of depressive symptoms, we applied Poisson regression models with robust variance estimators. Given the high prevalence of depressive symptoms in the study population, we used the Poisson log-link function to estimate prevalence ratios (PRs) rather than odds ratios, as the latter tend to overestimate the magnitude of the association when the outcome is common (prevalence > 10%) [29].

The primary analyses modeled triglyceride and total cholesterol concentrations as continuous variables. Restricted cubic splines with three knots (10th, 50th, and 90th percentiles) were used to allow for potential non-linear relationships, which were formally tested with Wald statistics. When no evidence of non-linearity was found, associations were summarized with linear models, reporting PRs per 10 mg/dL increment. For clinical interpretability, we also estimated contrasts at pre-specified values (200 vs. 120 mg/dL for triglycerides and 200 vs. 160 mg/dL for total cholesterol). As a secondary analysis, both lipids were additionally categorized according to guideline cutoffs (≥150 mg/dL for triglycerides and ≥200 mg/dL for total cholesterol) to facilitate comparison with clinically defined thresholds.

Since the magnitude of the association could be influenced by sex and overweight/obesity status, we evaluated effect modification. For multiplicative interaction, categorical versions of triglycerides, total cholesterol, and BMI were used (e.g., high vs. normal) to enhance clinical interpretability. For additive interaction, we assessed the departure from additivity between lipids and BMI using the relative excess risk due to interaction (RERI), the attributable proportion due to interaction (AP), and the synergy index (SI), with 95% confidence intervals estimated via the delta method [30].

All models were adjusted for potential confounding factors. The selection of these variables was guided by directed acyclic graphs (DAGs) [31,32]. The minimally sufficient adjustment set included age, sex, smoking, alcohol consumption, academic year, and household income (Figure S2). In models restricted to women, additional adjustment was performed for hormonal contraceptive use. To evaluate potential multicollinearity among covariates, we calculated variance inflation factors (VIFs). The mean VIF was 1.4, and no individual VIF exceeded 2, indicating that collinearity was not a major concern in our models.

Statistical significance for hypothesis testing and models was set at a *p*-value < 0.05. Interaction terms were evaluated on both multiplicative and additive scales, with inference for additive measures (RERI, AP, S) based on 95% confidence intervals (null = 0 for RERI/AP, null = 1 for S). All analyses were performed using STATA software, version 19.5 (StataCorp, College Station, TX, USA).

## 3. Results

Table 1 presents the sociodemographic, lifestyle, and clinical characteristics of the 219 participating medical students. Overall, most participants were women (62.6%) and between 18 and 23 years of age (76.7%). Most were single (94.1%), lived with their parents (85.8%), and were not employed (93.2%). Regarding socioeconomic status, 79.0% reported a monthly household income of $ 543.75 or more, and 86.8% did not receive a scholarship. In terms of lifestyle, 27.8% reported regular alcohol consumption, and 17.8% smoked cigarettes. Clinically, 68.9% had a normal BMI, only 5.5% reported chronic medical conditions (HIV, asthma, and arthritis being the most common), 9.6% reported a parental history of depression, and 23.7% reported parental overweight or obesity. Among women, 19.6% reported oral hormonal contraceptive use, and 28.8% reported menstrual irregularities (Table 1).

Overall, the median triglyceride concentration was 138 mg/dL (IQR 63), and 41.1% of participants had hypertriglyceridemia (defined as a concentration ≥ 150 mg/dL). Depressive symptoms were more frequent among those with elevated triglycerides compared to those with normal levels. Regarding total cholesterol, participants with depressive symptoms exhibited higher values, and hypercholesterolemia (≥200 mg/dL) was more frequent among them (56.5% vs. 34.1%; Table 2).

**Figure 1:**
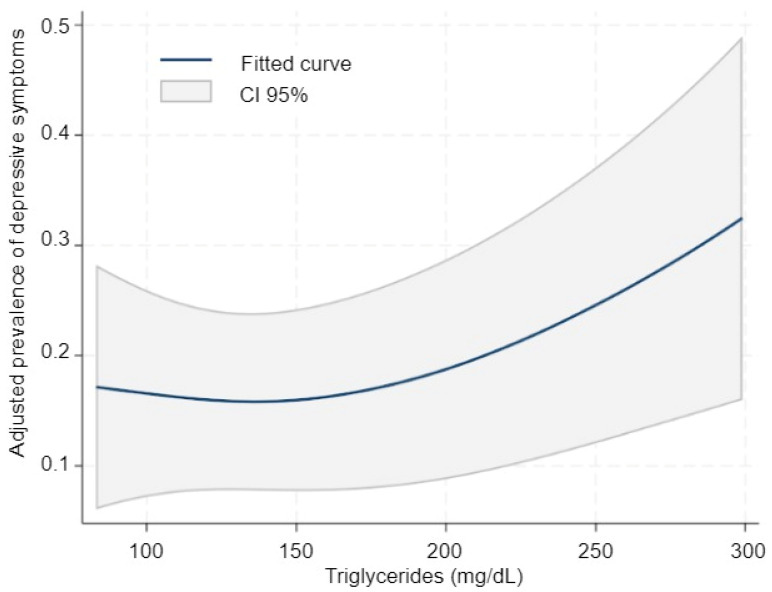
Adjusted prevalence of depression according to triglyceride levels (continuous modeling). Restricted cubic spline model showing the adjusted prevalence of depression across triglyceride concentrations. The solid line represents the fitted curve, and the shaded area indicates 95% confidence intervals. Models were adjusted for age, sex, smoking, alcohol consumption, academic year, and household income.

In the analysis using restricted cubic splines, the relationship between triglycerides and depression showed an ascending trend but was overall compatible with an approximately linear pattern (Figure 1). The joint Wald test of the spline terms did not provide evidence of non-linearity (χ^2^(1) = 2.06; *p* = 0.151), and therefore a more parsimonious adjusted linear model was reported. In this model, each 10 mg/dL increase in triglycerides was associated with a higher prevalence of depression (PR = 1.04; 95% CI 1.03–1.06; *p* < 0.001). To illustrate the magnitude of this association, comparing 200 mg/dL with 120 mg/dL yielded a PR of 1.39 (95% CI 1.22–1.59), consistent with the adjusted prevalences estimated by margins (0.44 vs. 0.32, respectively; see Table S1).

**Figure 2:**
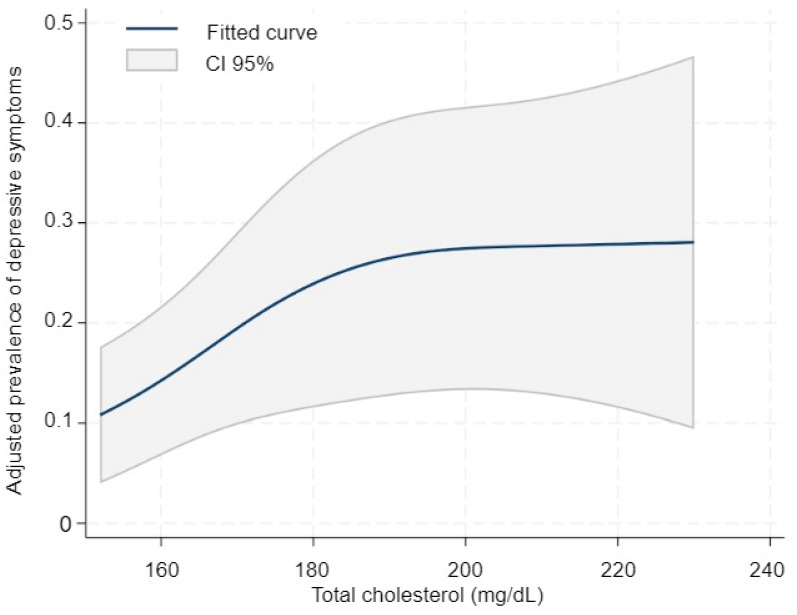
Adjusted prevalence of depression according to total cholesterol levels (continuous modeling). Legend: Restricted cubic spline model showing the adjusted prevalence of depression across total cholesterol concentrations. The solid line represents the fitted curve, and the shaded area indicates 95% confidence intervals. Models were adjusted for age, sex, smoking, alcohol consumption, academic year, and household income.

For total cholesterol, the restricted cubic spline analysis revealed a gentle increasing pattern, with some suggestion of flattening at higher concentrations (Figure 2). The joint Wald test of the spline terms did not reach conventional significance (χ^2^(1) = 3.15; *p* = 0.076), suggesting no substantial departure from linearity. Therefore, we reported a parsimonious linear model in which a 10 mg/dL increase in total cholesterol was associated with a higher adjusted prevalence of depression (PR 1.13; 95% CI 1.05–1.21; *p* = 0.001). In a comparison of clinically relevant values, 200 mg/dL versus 160 mg/dL was associated with a PR of 1.60 (95% CI, 1.20–2.14; *p* = 0.001), consistent with the trend suggested by the spline curve (Table S2).

In addition to the continuous analyses, we also examined triglycerides and total cholesterol as categorical variables using guideline-based cutoffs. After adjustment for potential confounders, students with elevated triglyceride levels (≥150 mg/dL) and those with elevated total cholesterol (≥200 mg/dL) had a higher prevalence of depressive symptoms compared with their counterparts with lower levels (PR = 1.76; 95% CI 1.24–2.48; *p* = 0.001, and PR = 1.66; 95% CI 1.19–2.31; *p* = 0.003, respectively). The crude models showed associations in the same direction (Table 3).

When examining potential effect modification by BMI, the association between triglycerides and depression appeared somewhat stronger among students with overweight or obesity compared to those with normal weight. However, the confidence intervals for the stratified estimates were wide and largely overlapping, and the formal test for interaction on the multiplicative scale was not statistically significant (see Table 4, panel A). Similarly, indices of interaction on the additive scale (RERI, AP, IS) were all close to null values, suggesting no meaningful departure from additivity. Taken together, these findings suggest that BMI did not materially modify the association between triglycerides and depressive symptoms in this sample (see Table 4, panel B). Patterns were similar when evaluating total cholesterol, with no evidence of effect modification by BMI on either the multiplicative (see Table 5, panel A) or additive scale (see Table 5, panel B).

Additionally, we examined whether sex modified the associations of interest. Elevated triglycerides (≥150 mg/dL) were strongly associated with depressive symptoms in men (PR = 3.01, 95% CI 1.14–7.93) and more modestly in women (PR = 1.50, 95% CI 1.06–2.13). The multiplicative interaction was statistically significant (*p* = 0.019; Table 6, panel A), although additive-scale indices (RERI, AP, S) were close to the null (Table 6, panel B). For total cholesterol, elevated levels (≥200 mg/dL) were associated with a higher prevalence of depressive symptoms in women (PR = 1.59, 95% CI 1.12–2.26) but not in men (PR = 2.00, 95% CI 0.81–4.92). However, the test for multiplicative interaction was not significant (*p* = 0.834; Table 7, panel A), and additive measures again indicated no departure from additivity (Table 7, panel B).

## 4. Discussion

In our study, elevated triglyceride and total cholesterol levels were independently associated with depressive symptoms among medical students. These findings emphasize the potential role of dyslipidemias not only as established risk factors for cardiovascular disease but also as factors potentially implicated in mental health problems. Given that medical students are particularly vulnerable to stress, academic pressure, and emotional burden, our results suggest that lipid alterations may have broader implications for both their physical and psychological well-being.

The prevalence of depressive symptoms identified in our study was comparable to that reported in Mexican university populations before the COVID-19 pandemic, when estimates ranged between 20% and 37% [18,19]. In contrast, studies conducted during the pandemic documented substantially higher prevalences, with figures of up to 45% [31] Our results also fall within the international range reported among medical students, which varies widely, from 28% to 48% [7,8]. These discrepancies could be attributed to several methodological differences, including the instrument used to assess depression, sample size, the academic year of the participants, and their sociodemographic characteristics. Despite these variations, the findings consistently reaffirm that depression remains a significant and persistent mental health problem among medical students worldwide.

Evidence on dyslipidemia among medical students is particularly scarce. In this population, up to 41.8% have been reported to present low levels of high-density lipoprotein cholesterol (HDL-C), a key component of metabolic syndrome [12]. Although not directly comparable to our findings on total cholesterol and triglycerides, this result underscores the importance of lipid abnormalities in young adults pursuing medical training.

Our continuous analyses showed that higher triglyceride and total cholesterol levels were associated with a greater prevalence of depressive symptoms, with approximately linear trends. Analyses using clinical cutoffs confirmed these findings, reinforcing the robustness of the associations. These results suggest that even moderate increases in lipid concentrations may be associated with the mental health of young adults. The fact that these associations were observed among medical students, who are expected to have greater awareness of cardiovascular risk factors, underscores the need to strengthen health promotion strategies within this population.

In our study, the prevalence of elevated triglyceride levels (41.1%) was higher compared with that of elevated total cholesterol levels (21.0%). This finding is consistent with previous reports showing that hypertriglyceridemia is more common than hypercholesterolemia among young adults in Mexico [33]. Comparable patterns have also been observed in Yemeni undergraduate students, Mexican-American young adults in the United States [34], and in Korean young adults, where national survey data indicate that high triglyceride levels are persistent in this age group [35]. One possible explanation is that triglyceride levels are more sensitive to lifestyle factors, such as dietary habits and physical inactivity [36], which are highly prevalent in the university population. Furthermore, obesity and insulin resistance, which contribute to elevated triglycerides, tend to manifest at an earlier age than conditions that raise total cholesterol levels [37,38], explaining the higher proportion observed in our sample. In this regard, it is noteworthy that 31% of our participants presented overweight or obesity, and 25% reported low physical activity, which may have contributed to the high prevalence of hypertriglyceridemia.

Our results showed that both elevated triglyceride and total cholesterol levels were significantly associated with a higher prevalence of depressive symptoms in the study sample. These findings suggest that alterations in the lipid profile may play a significant role in the mental health of young adults, even in populations such as medical professionals. Our findings are consistent with a recent meta-analysis that found significantly higher serum triglyceride concentrations among individuals with depression [39]. Furthermore, population-based studies in China [40], the United States [41,42], and the United Kingdom [43] have consistently shown that both hypertriglyceridemia and triglyceride-derived indices, such as the triglyceride-glucose (TyG) index or the TG/HDL-C ratio, are linked to an increased risk of depressive symptoms.

In contrast, prior evidence linking total cholesterol with depression remains inconsistent. Some longitudinal studies have reported that both persistently low and high levels of total cholesterol during adolescence are associated with an increased risk of depressive symptoms in early adulthood [44]. However, other investigations in hospitalized patients, older adults, and the general population have not confirmed this association [45,46,47], and some have even found a lower risk of depression among individuals with reduced total cholesterol levels [48]. Therefore, these findings suggest that the relationship between total cholesterol and depression is complex and may be influenced by age, health status, and methodological differences across studies.

Despite these inconsistencies, several biological mechanisms have been proposed to explain the link between lipid abnormalities and depression. Notably, the pathways through which triglycerides and total cholesterol may influence mental health appear to differ, suggesting that distinct metabolic and neurobiological processes underlie each association. Hypertriglyceridemia promotes a state of low-grade systemic inflammation, characterized by an increase in proinflammatory cytokines, such as interleukin-6 (IL-6), tumor necrosis factor-alpha (TNF-α), and C-reactive protein (CRP) [49]. These molecules cross the blood–brain barrier and negatively modulate serotonergic and dopaminergic neurotransmission, promoting the onset of depressive symptoms [50,51]. Furthermore, hypertriglyceridemia often coexists with insulin resistance, which alters glucose uptake in the central nervous system and generates an imbalance in brain energy availability, contributing to synaptic dysfunction [52]. Additionally, a high-calorie, high-fat diet, characteristic of young populations, promotes intestinal dysbiosis and the production of inflammatory metabolites that negatively impact the gut–brain axis [53]. Hence, these processes suggest that elevated triglycerides act as an early marker of metabolic and neuroinflammatory vulnerability associated with depression.

On the other hand, the mechanisms linking hypercholesterolemia to depression appear to differ from those described for triglycerides. Cholesterol plays a fundamental structural role in neuronal membranes, regulating their fluidity and the function of serotonin receptors and transporters. However, excess total cholesterol can alter these processes and compromise synaptic plasticity [54]. Of particular concern are cholesterol oxidation products, known as oxysterols, which cross the blood–brain barrier and have neurotoxic and proinflammatory effects, contributing to oxidative stress in the brain [55]. At the vascular level, hypercholesterolemia is associated with endothelial dysfunction and subclinical atherosclerosis, which can reduce cerebral perfusion and affect regions involved in mood regulation, such as the hippocampus and prefrontal cortex [56]. It has also been suggested that high total cholesterol levels influence the dysregulation of the hypothalamic–pituitary–adrenal axis, increasing vulnerability to stress and, consequently, to depression [57]. These mechanisms reinforce the idea that high total cholesterol impacts mental health through neurostructural, vascular, and endocrine pathways, distinct from but complementary to those observed in hypertriglyceridemia.

In our stratified analyses, we explored whether BMI and sex modified the associations between lipid levels and depressive symptoms. Although prevalence ratios tended to be higher among individuals with overweight/obesity and among men with elevated triglycerides, the formal tests for interaction on both the multiplicative and additive scales did not support significant effect modification. These analyses should therefore be interpreted as exploratory, particularly given the small size of some strata and the resulting wide confidence intervals. Nonetheless, the previous literature suggests that adiposity and sex-related biological differences could plausibly influence these associations. Obesity has been linked to depression through pathways involving insulin resistance, systemic inflammation, and dysregulation of lipid metabolism [58]. In contrast, hormonal differences, such as estrogen’s role in lipid and serotonergic pathways, may partly explain sex-specific patterns reported in other studies [59,60]. Hence, while our results did not provide statistical evidence of interaction, the observed tendencies align with mechanistic hypotheses that warrant further investigation in larger samples.

### Limitations

For a proper interpretation of our results, several factors must be taken into consideration. As in previous studies, the cross-sectional design of this analysis did not allow for establishing the temporal sequence between exposure and outcome; therefore, the estimated associations cannot be interpreted as causal. Information on depressive symptoms and covariates was obtained through self-report; however, we used instruments validated in the Mexican population to assess depression, diet, and physical activity, ensuring data accuracy. Both the questionnaires and the biochemical and anthropometric measurements were administered by trained and standardized personnel. Thus, differential misclassification bias is unlikely to occur.

Because triglyceride and total cholesterol levels were assessed using a portable monitor, clinical diagnoses of hypercholesterolemia or hypertriglyceridemia could not be established. Nevertheless, this approach is common in field-based epidemiological studies and is considered appropriate for population classification. Additionally, we did not determine the HDL and LDL cholesterol fractions, which limited a more detailed assessment of lipid profile alterations. Although body mass index was included as a covariate, a more precise measure of central adiposity, such as waist circumference, would have provided a better proxy for metabolic risk. The sample size may also have limited our statistical power to detect interactions; hence, effect modification analyses should be interpreted with caution. In addition, the relatively small number of depression cases within some strata (e.g., men with high triglyceride levels) may have produced unstable estimates, occasionally yielding inflated prevalence ratios with wide confidence intervals.

Finally, our study was conducted among medical students, a population characterized by specific academic demands and health conditions. Therefore, extrapolation should be restricted to university groups with comparable characteristics. Nevertheless, the potential biological mechanism linking dyslipidemia with depressive symptoms strengthens the external validity of our findings. It supports the plausibility that the observed associations could be replicated in similar settings.

## 5. Conclusions

The results of our study showed that elevated triglyceride and total cholesterol levels were independently associated with the presence of depressive symptoms in medical students. These findings suggest that alterations in lipid profiles may constitute a relevant biological factor in the mental health of young undergraduate students, adding to the previously documented psychosocial determinants. The identification of this association in a particularly vulnerable population highlights the need to integrate the assessment and promotion of metabolic health into institutional strategies for the prevention and management of depression. Furthermore, the evidence obtained provides a basis for future longitudinal research to clarify causal mechanisms and explore interventions aimed at reducing both cardiovascular risk and the impact of depression in this population. Promoting mental and metabolic health in medical students is not only essential for their own well-being but also to ensure that, in their future professional practice, they can provide comprehensive, high-quality care to their patients.

## Figures and Tables

**Table 1:** Select characteristics of participants.

Features	*n* = 219
	f (%)
Sex	
Woman	137 (62.6)
Man	82 (37.4)
Age	
18–20 years	90 (41.1)
21–23 years	78 (35.6)
≥24 years	51 (23.3)
Marital status	
No partner	206 (94.1)
With partner	13 (5.9)
Family monthly income	
<543 American dollars	46 (21.0)
≥543 American dollars	173 (79.0)
Living arrangements	
Living with parents	188 (85.8)
Living away from the family home	31 (14.2)
Remunerated employment	
Yes	15 (6.8)
No	204 (93.2)
Year in program	
First	71 (32.4)
Second	38 (17.4)
Third	43 (19.6)
Fourth	32 (14.6)
Fifth	35 (16.0)
Scholarship	
Yes	29 (13.2)
No	190 (86.8)
Regular alcohol consumption	
Yes	61 (27.8)
No	158 (72.2)
Regular cigarette smoking	
Yes	39 (17.8)
No	180 (82.2)
Body Mass Index categories	
Normal weight (18.5–24.9)	151 (68.9)
Overweight (25.0–29.9)	56 (25.6)
Obesity (≥30.0)	12 (5.5)
Chronic medical conditions	
Yes	12 (5.5)
No	207 (94.5)
Parental history of depression (any parent)	
Yes	21 (9.6)
No	198 (90.4)
Parental overweight/obesity (any parent)	
Yes	52 (23.7)
No	167 (76.3)
Hormonal contraceptive use (women only).	
Yes	43 (19.6)
No	176 (80.4)
Menstrual irregularities	
Yes	63 (28.8)
No	156 (71.2)

Abbreviations: f, frequency.

**Table 2:** Biochemical profile of participants according to the presence of depressive symptoms.

Variables	Total (*n* = 219)	Depressive Symptoms		
		No (*n* = 134)	Yes (*n* = 85)	*p*-Value ^a^
Triglycerides, mg/dL				
Median (IQR)	138 (63)	128.5 (73)	163 (120)	0.011
Triglycerides ≥ 150 mg/dL, f (%)				
Yes	90 (41.1)	42 (46.7)	48 (53.3)	0.001
No	129 (58.9)	92 (71.3)	37 (28.7)	
Total cholesterol, mg/dL				
Median (IQR)	172 (33)	167 (26)	180 (40)	0.001
Total cholesterol ≥ 200 mg/dL, f (%)				
Yes	46 (21.0)	20 (43.5)	26 (56.5)	0.006
No	173 (79.0)	114 (65.9)	59 (34.1)	

^a^ Comparing subjects by depressive symptoms status using Pearson’s chi-squared test for categorical variables and the Mann–Whitney U test for the difference in medians.

**Table 3:** Crude and adjusted prevalence ratios for the association between lipid profile and depressive symptoms.

Lipid Profile	Depressive Symptoms			
	PR (95% CI)	Valor-*p*	PR ^a^ (95% CI)	Valor-*p*
Triglycerides				
<150 mg/dL	Ref.		Ref.	
≥150 mg/dL	1.86 (1.33, 2.59)	<0.001	1.76 (1.24, 2.48)	0.001
Total Cholesterol				
<200 mg/dL	Ref.		Ref.	
≥200 mg/dL	1.66 (1.19, 2.30)	0.003	1.66 (1.19, 2.31)	0.003

Abbreviations: PR, prevalence ratio; CI, confidence interval; Ref., reference. ^a^ Models were adjusted for age, sex, smoking, alcohol consumption, academic year, and household income.

**Table 4:** Association between triglycerides and depressive symptoms, stratified by body mass index and tested for interaction.

Panel A. Multiplicative scale			
**Triglycerides**	**BMI < 25 kg/m^2^** **(Normal)**	**BMI ≥ 25 kg/m^2^** **(Overweight/Obesity)**	**Interaction *p*-Value**
	**PR (95% CI) ^a^, *p*-value**	**PR (95% CI) ^a^, *p*-value**	
<150 mg/dL	Ref.	Ref.	—
≥150 mg/dL	1.53 (1.01–2.32), *p* = 0.043	1.90 (0.86–4.16), *p* = 0.109	0.582
Panel B. Additive scale			
**Measure**	**Estimate**	**95% CI**	***p*-Value**
RERI	0.23	−0.67 to 1.14	0.611
AP	0.13	−0.36 to 0.63	0.599
SI	1.45	−0.90 to 3.80	0.228

Abbreviations: BMI, body mass index; PR, prevalence ratio; RERI, relative excess risk due to interaction; AP, attributable proportion due to interaction; SI, synergy index. ^a^ Models were adjusted for age, sex, smoking, alcohol consumption, academic year, and household income. Panel A: PRs are derived from a single Poisson regression including the TG × BMI interaction term; interaction *p*-value corresponds to the joint test of the interaction. Panel B additive measures (RERI, AP, SI) with 95% CIs via the delta method (null = 0 for RERI/AP; null = 1 for IS).

**Table 5:** Association between total cholesterol and depressive symptoms, stratified by body mass index and tested for interaction.

Panel A. Multiplicative scale			
**Total Cholesterol**	**BMI < 25 kg/m^2^** **(Normal)**	**BMI ≥ 25 kg/m^2^** **(Overweight/Obesity)**	**Interaction *p*-Value**
	**PR (95% CI) ^a^, *p*-value**	**PR (95% CI) ^a^, *p*-value**	
<200 mg/dL	Ref.	Ref.	—
≥200 mg/dL	1.69 (1.03–2.79), *p* = 0.038	1.78 (0.99–3.23), *p* = 0.060	0.287
Panel B. Additive scale			
**Measure**	**Estimate**	**95% CI**	***p*-Value**
RERI	0.04	−0.93 to 1.01	0.934
AP	0.03	−0.58 to 0.63	0.933
SI	1.07	−0.79 to 2.94	0.258

Abbreviations: BMI, body mass index; PR, prevalence ratio; RERI, relative excess risk due to interaction; AP, attributable proportion due to interaction; SI, synergy index. ^a^ Models were adjusted for age, sex, smoking, alcohol consumption, academic year, and household income. Panel A PRs derived from a single Poisson model, including the TC × BMI interaction term; interaction *p*-value from the joint test of the interaction. Panel B additive measures (RERI, AP, SI) with 95% CIs via the delta method (null = 0 for RERI/AP; null = 1 for IS).

**Table 6:** Association between triglycerides and depressive symptoms, stratified by sex and tested for interaction.

Panel A. Multiplicative scale			
**Triglycerides**	**Man**	**Women**	**Interaction *p*-Value**
	**PR (95% CI) ^a^, *p*-value**	**PR (95% CI) ^b^, *p*-value**	
<150 mg/dL	Ref.	Ref.	—
≥150 mg/dL	3.01 (1.14–7.93), *p* = 0.026	1.50 (1.06–2.13), *p* = 0.020	0.019
Panel B. Additive scale			
**Measure**	**Estimate**	**95% CI**	***p*-Value**
RERI	−1.71	−4.66–1.25	0.258
AP	−0.35	−0.87–0.17	0.189
SI	0.69	0.38–1.01	<0.001

Abbreviations: BMI, body mass index; PR, prevalence ratio; RERI, relative excess risk due to interaction; AP, attributable proportion due to interaction; SI, synergy index. ^a^ Models were adjusted for age, sex, smoking, alcohol consumption, academic year, and household income. ^b^ Models were also adjusted for hormonal contraceptive use. Panel A PRs derived from a single Poisson model, including the TG × BMI interaction term; interaction *p*-value from the joint test of the interaction. Panel B additive measures (RERI, AP, SI) with 95% CIs via the delta method (null = 0 for RERI/AP; null = 1 for IS).

**Table 7:** Association between total cholesterol and depressive symptoms, stratified by sex and tested for interaction.

Panel A. Multiplicative scale			
**Total Cholesterol**	**Man**	**Women**	**Interaction *p*-Value**
	**PR (95% CI) ^a^, *p*-Value**	**PR (95% CI) ^b^, *p*-Value**	
<200 mg/dL	Ref.	Ref.	—
≥200 mg/dL	2.0 (0.81–4.92), *p* = 0.132	1.59 (1.12–2.26), *p* = 0.009	0.834
Panel B. Additive scale			
**Measure**	**Estimate**	**95% CI**	***p*-Value**
RERI	−0.20	−2.35–1.95	0.855
AP	−0.05	−0.61–0.50	0.855
SI	0.93	0.28–1.59	0.845

Abbreviations: BMI, body mass index; PR, prevalence ratio; RERI, relative excess risk due to interaction; AP, attributable proportion due to interaction; SI, synergy index. ^a^ Models were adjusted for age, sex, smoking, alcohol consumption, academic year, and household income. ^b^ Models were also adjusted for hormonal contraceptive use. Panel A PRs derived from a single Poisson model, including the TG × BMI interaction term; interaction *p*-value from the joint test of the interaction. Panel B additive measures (RERI, AP, SI) with 95% CIs via the delta method (null = 0 for RERI/AP; null = 1 for IS).

## Data Availability

The data that support the findings of this study are openly available in Mendeley Data at https://doi.org/10.17632/c6ymfd7sbv.1.

## Supplementary Materials

The following supporting information can be downloaded at https://www.mdpi.com/article/10.3390/diseases13100326/s1: Figure S1: Participants’ selection flowchart; Figure S2: Directed acyclic graph for the association between total cholesterol and triglycerides with depressive symptoms; Table S1: Association of Triglyceride Levels with Depression: Tests of Linearity and Clinical Contrasts; Table S2: Association of Total Cholesterol with Depression: Linearity and Clinical Contrasts

## Abbreviations

The following abbreviations are used in this manuscript:

WHO	World Health Organization
HDL-C	High-Density Lipoprotein Cholesterol’
BMI	Body Mass Index
ENSANUT	the National Health and Nutrition Survey (Encuesta Nacional de Salud y Nutrición),
CESD-7	Center for Epidemiologic Studies Depression Scale
IPAQ-7	International Physical Activity Questionnaire
RP	Ratio prevalence
CI	Confidence interval
DAGs	Directed acyclic graphs
