# Supplementary material for: Triglycerides, Cholesterol, and Depressive Symptoms Among Undergraduate Medical Students: A Cross-Sectional Study
Source: Diseases. 2025 Oct 2;13(10):326. doi: 10.3390/diseases13100326 (PMC12562626; doi:10.3390/diseases13100326)

Figure S2: Directed acyclic graph for the association between cholesterol and triglycerides with depressive symptoms.

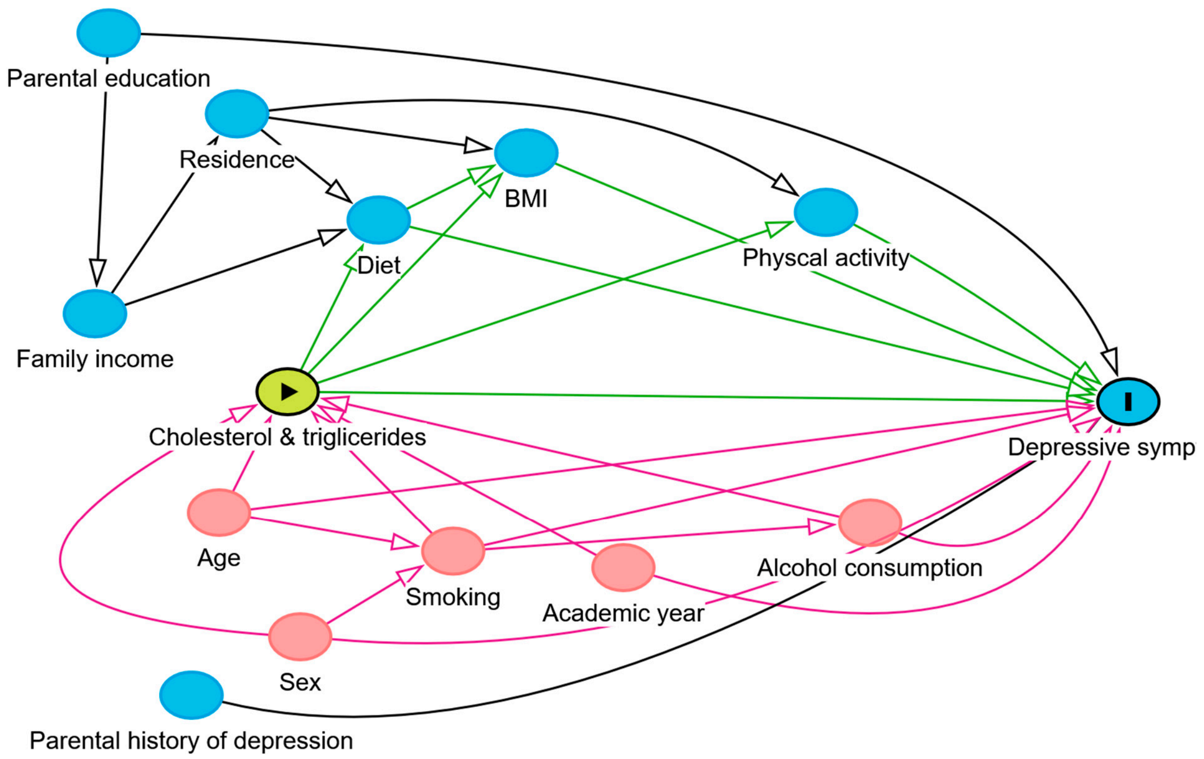

Supplement: Supplementary file 1 [file diseases-13-00326-s001.zip › Supplementary Figure S2.pdf]
