# Supplementary material for: Triglycerides, Cholesterol, and Depressive Symptoms Among Undergraduate Medical Students: A Cross-Sectional Study
Source: Diseases. 2025 Oct 2;13(10):326. doi: 10.3390/diseases13100326 (PMC12562626; doi:10.3390/diseases13100326)
Supplement: Supplementary file 1 [file diseases-13-00326-s001.zip › Tabla S1.pdf]

Table S1. Association of Triglyceride Levels with Depression: Tests of Linearity and Clinical Contrasts

Analysis		Estimate	95% CI	p-value
Wald test for non-linearity ($\chi^2(1)$)		2.06	—	0.151
Linear model (per +10 mg/dL)		PR = 1.04	1.03 – 1.06	<0.001
Clinical contrast (200 vs 120 mg/dL)		PR = 1.39	1.22 – 1.59	<0.001
Adjusted prevalence (120 mg/dL)		0.32	0.26 – 0.38	—
Adjusted prevalence (200 mg/dL)		0.44	0.38 – 0.51	—

PR: prevalence ratio; CI: confidence interval.

Table S1 summarizes the main results of the analysis of triglycerides in relation to depression. Restricted cubic spline models suggested an ascending trend, but the Wald test did not provide evidence of non-linearity. Therefore, a linear model was reported. The table shows the test for non-linearity, the effect of triglycerides modeled as a continuous variable, and a clinically relevant contrast (200 vs 120 mg/dL), as well as adjusted prevalences at these two points.
