# Supplementary material for: Triglycerides, Cholesterol, and Depressive Symptoms Among Undergraduate Medical Students: A Cross-Sectional Study
Source: Diseases. 2025 Oct 2;13(10):326. doi: 10.3390/diseases13100326 (PMC12562626; doi:10.3390/diseases13100326)
Supplement: Supplementary file 1 [file diseases-13-00326-s001.zip › Table S2.pdf]

Table S2. Association of Total Cholesterol with Depression: Linearity and Clinical Contrasts

Analysis	Estimate	95% CI	p-value
Wald test for non-linearity ($\chi^2(1)$)	3.15	—	0.076
Linear model (per +10 mg/dL)	PR = 1.13	1.05 – 1.21	0.001
Clinical contrast (200 vs 160 mg/dL)	PR = 1.60	1.20 – 2.14	0.001

PR: prevalence ratio; CI: confidence interval.

Table S2 summarizes the main results of the analysis of total cholesterol in relation to depression. Restricted cubic spline models suggested an increasing trend with some flattening at higher concentrations, but the Wald test did not provide strong evidence of non-linearity. Therefore, the results are summarized using a linear model, along with a clinically relevant contrast (200 vs. 160 mg/dL).
