# Supplementary figures and images for: Triglycerides, Cholesterol, and Depressive Symptoms Among Undergraduate Medical Students: A Cross-Sectional Study
Source: Diseases. 2025 Oct 2;13(10):326. doi: 10.3390/diseases13100326 (PMC12562626; doi:10.3390/diseases13100326)

**Figure S1. Participants' selection flowchart.**

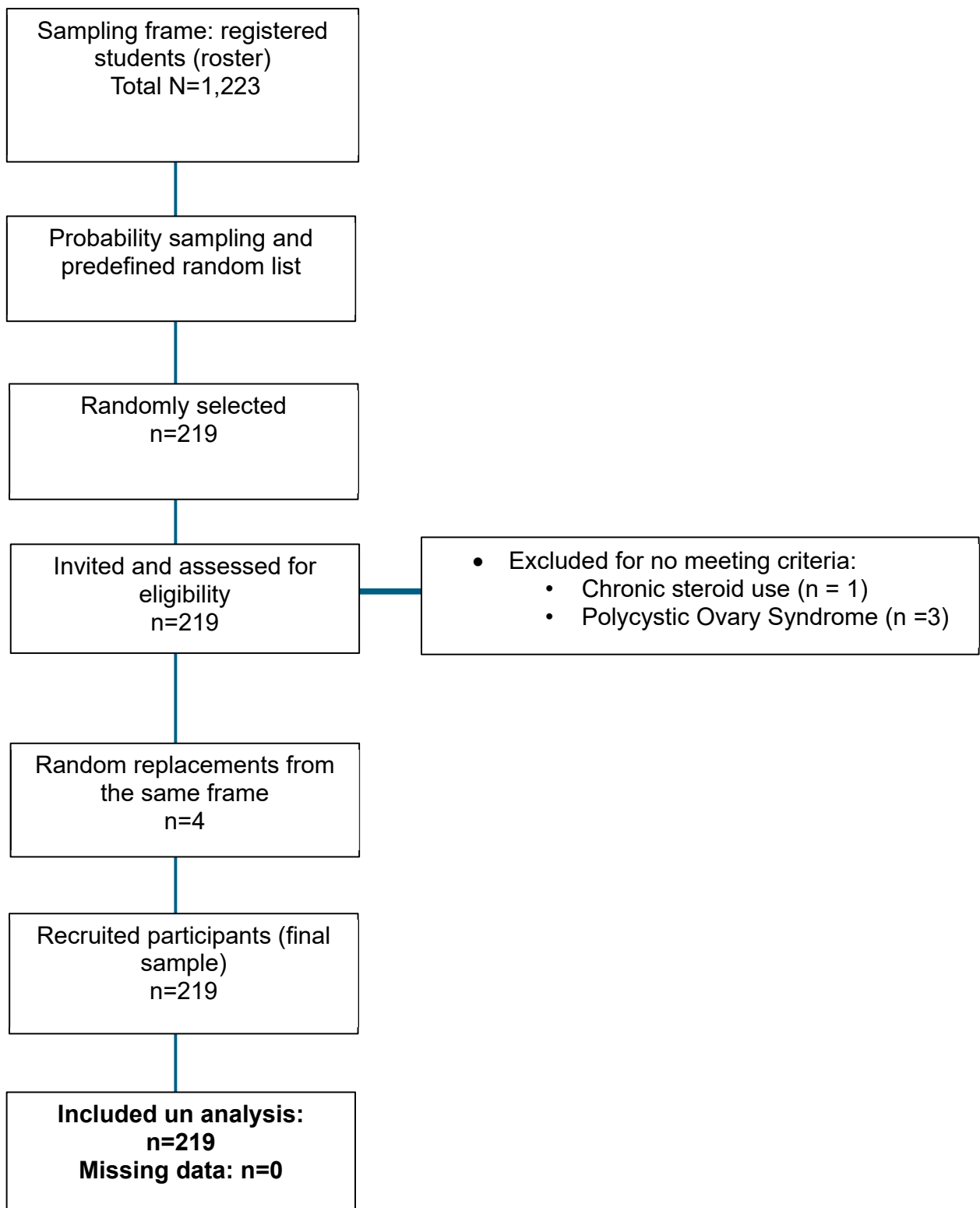

Supplement: Supplementary file 1 [file diseases-13-00326-s001.zip › Supplementary Figure S1_.pdf]
